# Methodological Considerations Regarding “Prevalence and Associated Risk Factors of Suicidal Tendencies Among Bangladeshi Public University Students”

**DOI:** 10.1002/hsr2.73291

**Published:** 2026-09-23

**Authors:** Abdullah Tahir

**Affiliations:** ^1^ Ayub Medical College Abbottabad Pakistan

Dear Editor,

The research by Uzzal and colleagues that evaluated the prevalence and risk factors for the occurrence of self‐harm among university students in Bangladesh is a very interesting publication [1]. This research used a mixed approach (cross‐sectional survey and in‐depth interviews) to assess a significant public health issue in a location with limited access to high‐quality epidemiological data. The results of this study contribute important context‐specific information and highlight the critical need to increase strategies for improving campus‐based mental health services.

Suicide ideation and related behaviors were assessed through self‐report measures, and multivariable logistic regression was used to identify variables independently associated with suicidal tendencies. The authors reported a high prevalence of suicidal ideation and significant associations with the female sex, history of suicide in the family, various mental health symptoms, substance use, and stressors within the psychosocial realm.

The study generated valuable descriptive data; a few methodological considerations, however, warrant discussion. Since this was a cross‐sectional study design, temporal inference, or causal relation (how long ago exposure and outcome occurred) cannot be determined because both exposure and outcome were collected simultaneously. This is emphasized repeatedly in the current methodological literature [2]. Therefore, any risk factor that has been identified as a cause of suicidal ideation may actually be an associated or resultant factor and not an antecedent. Close attention to the semantic difference between association and causation may help avoid overinterpretation.

In addition, attention should be given to the sampling strategy and the extent to which it accurately reflects the population from which data were drawn. The means of gathering data were through voluntary online participation from selected public universities, which made the method a convenience sample. Non‐probability sampling can sometimes be a practical choice, but it can also introduce selection bias to results and restrict external validity [3]. Due to the potential for either high motivation to participate (leading to an increase in estimated prevalence rates) or low motivation to participate (leading to underestimation of burden), it is uncertain whether the findings have the potential to be transferred to other locations outside the sampled institutions. Contemporary recommendations regarding generalizability indicate that researchers should use clear definitions of their source population and pattern of participation to allow for appropriate interpretation [4].

There is some concern that the associations found in this study may be affected by residual confounding. Multivariable logistic regression was used to conduct statistical adjustments for ethnicity/race, education, and age, but was limited to measured and not unmeasured covariates, for example, either the severity of the subjects' depressive symptoms at baseline, or levels of socioeconomic adversity, or prior history of trauma could have affected both the development of suicidality and the elicited stressors (as an example only). Modern epidemiological studies suggest that uncontrolled confounding may distort both the strength (magnitude) and direction of observed relationships [5]. Addressing the potential for unmeasured confounding and making model assumptions explicit may help improve the clarity of the conclusions drawn from this study.

Finally, effect size interpretation needs attention as multiple significant associations are reported; using significance alone may not fully inform a reader of the clinical relevance. The current best practice is to use effect size and precision for interpretation and avoid reporting an arbitrary cut‐off. In cases where the odds ratios are modestly adjusted, it is important to interpret these odds ratios in their context so that readers do not misrepresent the degree of impact in practice. Reporting and discussing confidence intervals may help readers assess the stability of estimates and the possibility of imprecision [6].

The multiplicity of predictors should have been considered in the current analysis. As the number of predictor variables assessed increases, so does the likelihood of false‐positive findings [7, 8]. To improve statistical transparency, it would help to clarify whether the model specification was developed based on a hypothesis, explored and whether any shrinkage or correction method was used.

Measurement factors also need to be considered. Using self‐report to measure sensitive outcomes may introduce possible misclassification and social desirability bias. Research shows that survey modality and perceived anonymity can impact how many symptoms will be disclosed and how mental health symptoms are reported [9]. This limitation is especially important to note because of the stigma around mental health in many cultures.

The credibility of any research publication is enhanced by following recommended reporting standards. Detailed descriptions of how sample sizes were chosen, what definitions were used to operationalize variables, methods used to deal with missing data, and the analyses chosen for each aspect of the research allow replication and allow for comparisons between studies that use the same or similar methodological approaches [10]. Greater transparency in model diagnostics and in handling incomplete responses may further help align studies with current expectations for reporting observational research [11].

Despite these limitations, Uzzal et al. have provided important evidence [1] of the extent of psychological vulnerability among university students in Bangladesh. They advocate for the implementation of systematic screening at universities, access to campus counseling, and efforts to reduce upstream stressors. Future studies would also benefit from designs that address the limitations noted above.

In conclusion, Uzzal et al. have established themselves as important contributors to the body of research related to student mental health in South Asia. Adhering to the methodological recommendations mentioned above would strengthen the validity, clarity, and policy implications of their research.

## Author Contributions

**Abdullah Tahir:** writing – original draft, investigation, writing – review and editing, resources, supervision, methodology, visualization, project administration.

## Funding

The author has nothing to report.

## Conflicts of Interest

The author declares no conflicts of interest.

## Use of Artificial Intelligence

The authors declare that artificial intelligence (AI) tools were used in the preparation of this manuscript, including assistance with language editing and manuscript drafting. All AI‐assisted content was reviewed and verified by the authors, who take full responsibility for the accuracy and integrity of the work.

## Author Statement

All authors have read and approved the final version of the manuscript. Abdullah Tahir had full access to all of the data in this study and takes complete responsibility for the integrity of the data and the accuracy of the data analysis.

## Transparency Statement

Abdullah Tahir affirms that this manuscript is an honest, accurate, and transparent account of the study being reported; that no important aspects of the study have been omitted; and that any discrepancies from the study as planned have been explained.

## Data Availability

No new data were generated or analyzed in support of this research. The authors confirm that the data supporting the findings of this study are available within the article.
